# Language-controllable programmable metasurface empowered by large language models

**DOI:** 10.1515/nanoph-2023-0646

**Published:** 2024-01-04

**Authors:** Shengguo Hu, Jiawen Xu, Mingyi Li, Tie Jun Cui, Lianlin Li

**Affiliations:** State Key Laboratory of Advanced Optical Communication Systems and Networks, School of Electronics, Peking University, Beijing 100871, China; State Key Laboratory of Millimeter Waves, Southeast University, Nanjing 210096, China; Pazhou Laboratory (Huangpu), Guangzhou, Guangdong 510555, China

**Keywords:** programmable metasurface, large language models, EM manipulation

## Abstract

Programmable metasurface has become a prominent tool in various areas including control, communication, computing, and so on, due to its unique capability in the electromagnetic (EM) manipulation. However, it is lack of the intelligence in the sense that it usually requires the manual intervention, and thus makes it hard to behavior as the human process. To endow the programmable metasurface with the intelligence, we here proposed the concept of the language-controllable programmable metasurface for autonomous EM manipulations by exploring the notable capability of large language models (LLMs) in attaining the human-like intelligence. We have established a proof-of-principle system of language-controllable programmable metasurface, where, for illustration, the programmable metasurface is designed to have 32 × 24 binary electronically controllable meta-atoms and work at around 5.5 GHz. In addition, we have constructed a visual-semantic map to facilitate the language-controllable EM manipulation in three-dimensional (3D) physical environments. We have experimentally demonstrated that our language-controllable programmable metasurface is capable of decomposing autonomously an ambiguous task of EM manipulation into a sequence of executable ones and implementing them individually in real-world indoor settings. We expect that the presented strategy could hold promising potential in pushing programmable metasurfaces towards human-level autonomous agents, which are capable of accomplishing the smart EM-involved multi-modality manipulations through self-directed planning and actions.

## 1 Introduction

Programmable metasurface, an ultrathin engineered structure, has been well recognized as a prominent agent for the flexible electromagnetic (EM) manipulation [1–4], and has found its valuable applications in various areas, such as, control [5, 6], communication [7–9], computing [10], and so on [11–13]. Compared with its ancestors, i.e., earlier versions of metamaterials and metasurfaces [14, 15], the programmable metasurface exhibits a technology trend towards a self-organizing EM agent through the embedded active components [16], sensors [17] and algorithms [18]. Thereby, the programmable metasurface holds the promising capabilities in configuring and optimizing its configuration and operational parameters according to environment settings and user’s demands. However, it is lack of the intelligence in the sense that the control operations are realized from the manual inputs and trained with limited dataset in a relatively isolated environment, and the performance is largely dependent on predefined operational condition and corresponding configurations. In addition, the programmable metasurface is usually developed for solving dedicated simple problems, and is inefficient in dealing with emerging complex EM-related manipulation tasks. For instance, the so-called smart metasurface equipped with sensors may undertake some self-adaptive functions but the functions must be programmed and loaded in advance [19], which cannot directly represent the will of the operator in real time.

Recently, large language models (LLMs), for instance, generative pre-trained transformer (GPT) [20], bidirectional encoder representation from transformer (BERT) [21], large language model Meta AI (LLaMA) [22], Falcon LLM [23], to name a few, have achieved notable successes, demonstrating the remarkable potential in attaining the human-level intelligence [24]. These models are trained over the web-scale dataset through so-called self-learning strategy [25], and are enabled with the zero-shot inference, generative, conversation, common sense understanding, and prediction capabilities. Thereby, they are revolutionarily shaping the technologies to make them attain the human-level inference and decision-making capabilities [26–28]. For instance, HuggingGPT can interact with users, understand their goals and accomplish complex multimodality tasks by exploring the conversation capability of LLMs with external models on HuggingFace [29]. LMA3, an LLM-based self-driven agent developed by Colas et al., is capable of autonomously setting goals for itself, and gradually improving its capability by exploring the surrounding environment [30]. SayCan, developed by Ahn et al., focus on investigating a wide range of manipulation and navigation skills utilizing a mobile manipulator robot [31]. Of course, we here anticipate that the LLM can enable the programmable metasurface to understand and interact with the cross-modality environment, evolving towards a human-like autonomous EM agent. Furthermore, we expect that LLMs can enable programmable metasurfaces to enjoy the predictability feature, and hence, realize improved and proactive capabilities of dynamic EM manipulations in changing environments, such as, localization, beamforming, power allocation, cloaking, as well as, spectrum management, even for the unseen scenarios.

In this article, we have proposed the concept of the language-controllable programmable metasurface, which has the capability of accomplishing self-directed EM manipulation tasks like the human process. For this reason, we would like to refer to it as programmable metasurface agent. We have established a proof-of-principle, and demonstrated experimentally its capability of human-level EM manipulation in four aspects. First, it is capable of decomposing a relatively complex EM manipulation task into a sequence of simpler ones by employing the self-planning capability of LLMs, and implementing them individually. Second, it can explore the unknown environment, and continually refine the execution code of EM manipulation based on environment feedback through trial and error. Third, the actions are taken to communicate with the other agents or real humans for sharing information or collaboration. Then, we can envision that the presented programmable metasurface agent will become an incubator to form more future smart devices and systems.

## 2 System configuration and operational principle

**Figure 1:**
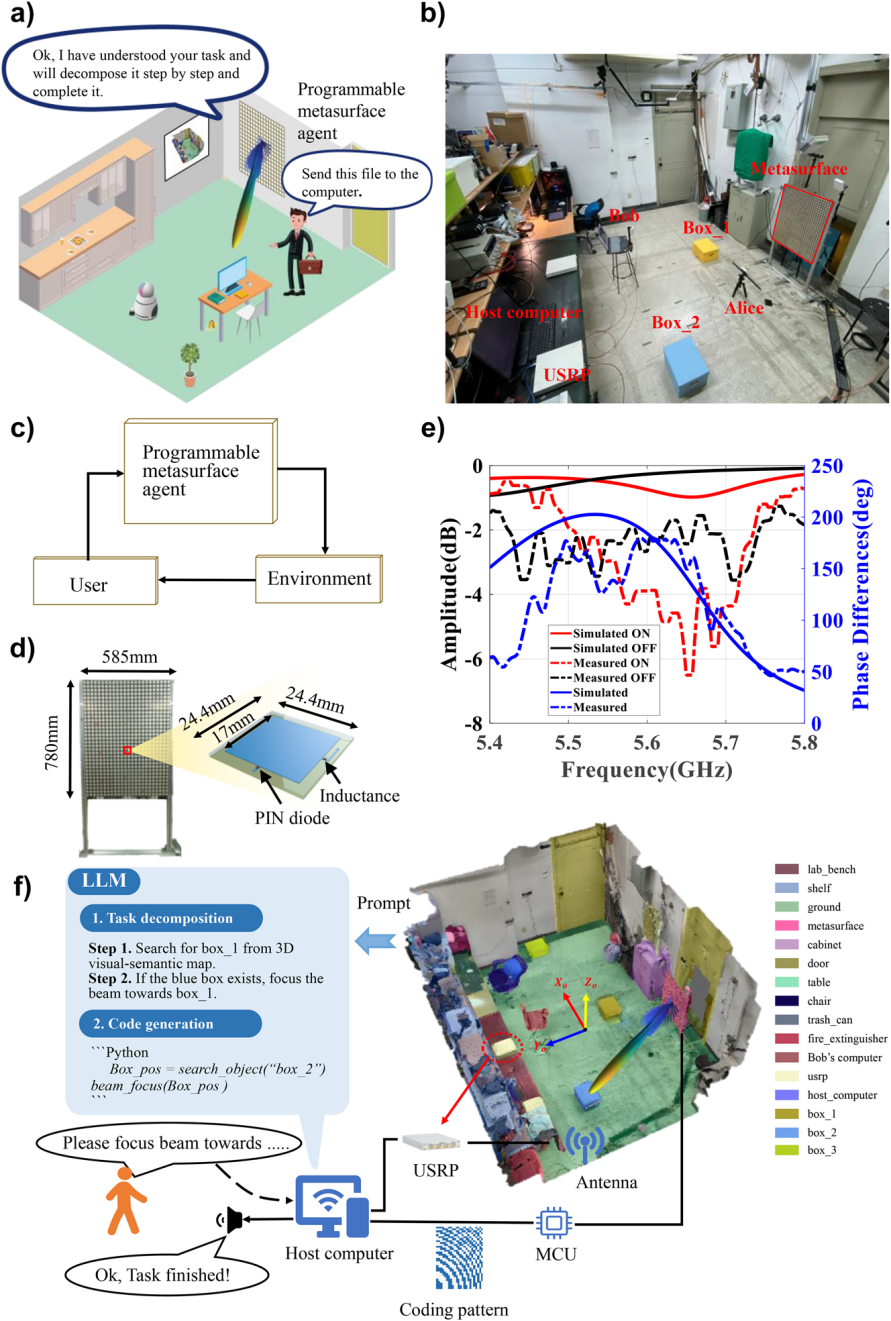
System configuration of the proposed programmable metasurface agent. (a, c) Conceptual illustration of programmable metasurface agent in indoor physical environment, where users are allowed to interact with the programmable metasurface agent through natural language voice or text. The programmable metasurface robot is capable of understanding the user’s intention, autonomous task planning and decomposition, and action (i.e., EM manipulations here) in 3D physical environment. (b) Photograph of experimental system configuration which consists of a programmable metasurface, MCU, host computer, USRP, and antennas. (d) Photograph of programmable metasurface with size of 780 mm × 560 mm, where some details of meta-atom have been provided as well. (e) The EM performance of the electronically-controllable meta-atoms numerically and experimentally (f) workflow of developed programmable metasurface agent. Here, a 3D visual-semantic map has been provided as well.

In this section, we will elaborate on the configuration and operation of the proposed language-controllable programmable metasurface. In a nutshell, one key motivation behind our strategy is to equip LLMs with crucial human-level decision-making capability to make the programmable metasurface behave like humans and complete various EM manipulation tasks effectively. For illustration purpose, the programmable metasurface agent is designed to serve as an artificial intelligent (AI) assistant in smart human-robot home scenarios who is mainly responsible for understanding the user’s language requests and accomplishing the intended EM manipulation tasks, as illustrated in Figure 1a. For example, when the metasurface robot receives the user’s request, e.g., “send this file to the computer”, it can automatically understand that task and decomposes it into a series of simple subtasks and implement them in a step-by-step way, as detailed below.

### 2.1 System configuration

Here, we discuss the system configuration of the proposed programmable metasurface agent in detail. As shown in Figure 1b and c, the whole system is composed of an LLM module as its central controller, a large-aperture programmable metasurface as the EM manipulation module in physical environment, and a host computer for the data postprocessing. In addition, a micro control unit (MCU) has been integrated into the programmable metasurface, which is responsible for converting the control coding sequence from the host computer to programmable metasurface, and a low-cost commercial software-defined radio device (Ettus USRP X310) has also been introduced for receiving feedback radio signals through connected antennas from surrounding environment. Here, both the USRP and metasurface communicate with the host computer via the Ethernet under the transmission control protocol (TCP). In our implementations, the programmable metasurface agent is designed to operate at around 5.5 GHz band, which could be readily scalable to other frequency bands. The programmable metasurface is composed of 32 × 24 binary electronically controllable meta-atoms with size of 0.780 m × 0.585 m, as shown in Figure 1d, where the meta-atom’s details are also reported. Note that the meta-atom has binary states, in the sense that the reflection phase response changes by 180° near 5.5 GHz, while the amplitude remains almost constant when the soldiered PIN diode is switched from OFF (ON) to ON (OFF). Here, the state of the PIN diode is controlled by an FPGA-based 50 MHz clock MCU. We have conducted numerical and experimental tests to examine the basic EM performance of the designed one-bit meta-atom, and the corresponding results have been presented in Figure 1e. Here, we have performed full-wave simulations using a commercial full-wave EM simulator (CST Microwave Transient Simulation Package 2017), and performed the experimental test directly in our lab environment. As shown in Figure 1e, we can readily observe that the reflected phase of the meta-atoms undergoes a 180° phase difference when the PIN diode is switched from ON (OFF) to OFF (ON) in the selected frequency range. Then, with massive above meta-atoms and suitable control coding sequences, we expect that the programmable metasurface agent is capable of autonomously solving a range of complex EM manipulation tasks. More details about the programmable metasurface can be found in Supplementary Note 1.

### 2.2 Workflow of language-controllable programmable metasurface

As mentioned previously, the programmable metasurface agent relies on two critical factors: (i) the powerful EM manipulation capability of programmable metasurface in physical environment, and (ii) the strong capabilities of inference, zero-sample learning, task decomposition, and code generation of LLMs in virtual digital world. Then, the integration of programmable metasurface with LLMs enables to achieve the autonomous EM manipulation and accomplish the complex tasks requested by the user. As mentioned above, a 3D visual-semantic map has been established to facilitate the EM manipulation in real-world physical environments using the language-controllable programmable metasurface. As shown in Figure 1f, in the 3D visual-semantic map, the whole environment has been semantically segmented and their semantics are marked in different colors, where the objects in the environment are all modeled as cubic, and each object was assigned a location with respect to the global coordinate system. For the efficiency consideration, the 3D visual-semantic map has been created in advance as the prompt for the LLM to search for the locations of intended subjects in the physical environment.

Now, we will elaborate on the four-step workflow of programmable metasurface agent, as shown in Figure 1f. First, a user can interact with the programmable metasurface in voice or text format through the LLMs installed in the host computer. For instance, when the LLM receives the user’s request, for instance, “please illuminate the box_2”, it will decompose the original ambiguous task into a sequence of simple but implementable subtasks, and then generate the implementable Python codes for subtasks. Second, the LLM will calculate the exact location of “box_2” in context of the created 3D visual-semantic map. Third, the host computer runs the Python code to generate the control coding sequence of metasurface such that the resultant radiation beam can be focused towards box_2. Finally, the host computer will respond to the user with a voice or text message that says, i.e., “Beam focus has been successfully completed.” It is worthy of mentioning that if the subject does not exist in the 3D visual-semantic map, the programmable metasurface agent can autonomously explore the unseen environment and continuously improve the execution Python code for the smart EM manipulation through the trial-and-error approach, as demonstrated in Section 3.3. To summarize, we can conclude that one can interact with the programmable metasurface agent in a free manner, enabling the autonomous EM manipulation in real-world settings.

## 3 Experimental results

In this section, we will experimentally demonstrate the autonomous EM manipulation capabilities of the developed programmable metasurface agent in four aspects. First, we will look into the language-controllable beam-focusing capability of the programmable metasurface agent. Afterwards, we will demonstrate the self-planning capability of the programmable metasurface agent in accomplishing complex EM manipulation tasks, and the active exploration ability in dealing with the unknown environment. Finally, we consider a relatively realistic scenario, i.e., wireless information sharing, to demonstrate the promising potential of programmable metasurface agent for the wireless chatting with other agents or human.

### 3.1 Results of the language-controllable beam focusing

**Figure 2:**
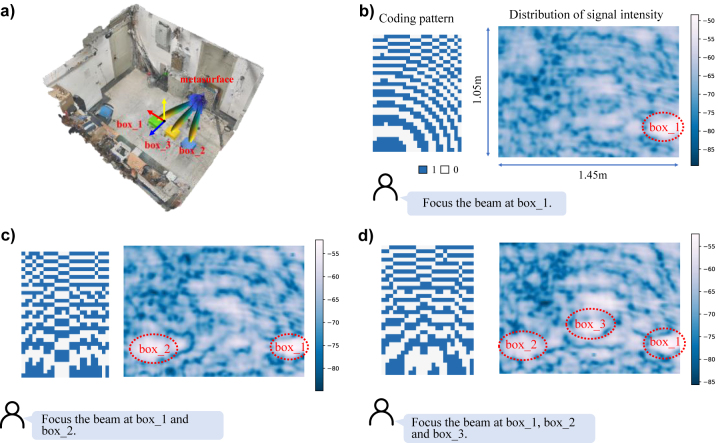
Experimental results of language-controllable beam focusing. (a) Experimental setup, (b) single-target focus scenario, (c) dual-target focus scenario, (d) triple-target focus scenario.

As the first set of fundamental examinations, we would like to provide some insights into the language-controllable EM beam manipulation of programmable metasurface agent, and corresponding experimental results have been reported in Figure 2. In our experiments, we have placed three boxes for the beam focusing targets in our lab environment (see Figure 2a), and ask the programmable metasurface agent to accomplish both single-target and multi-target beam focusing tasks when it receives language request, i.e., ‘please focus the beam towards Box-1’. Figure 2b reports the calculated control coding pattern [32] of the metasurface for single-target beam focusing in the left, and the corresponding 2D distribution of signal intensity in the right. Here, the 2D distribution has been achieved via the so-called plane scanning technique, where the scanning area with size of 1.45 m × 1.05 m is set as a plane parallel to the metasurface, and has the distance of 1.53 m away from the programmable metasurface. Further, we have considered the scenario for the dual target- and triple-target beam focusing tasks, and reported corresponding results in Figure 2c and d, respectively. Then, it can be readily observed that the programmable meatsurface agent is capable of allocating the wireless energy towards the intended targets in a language-controllable interaction manner.

### 3.2 Results of self-planning complex EM manipulation

**Figure 3:**
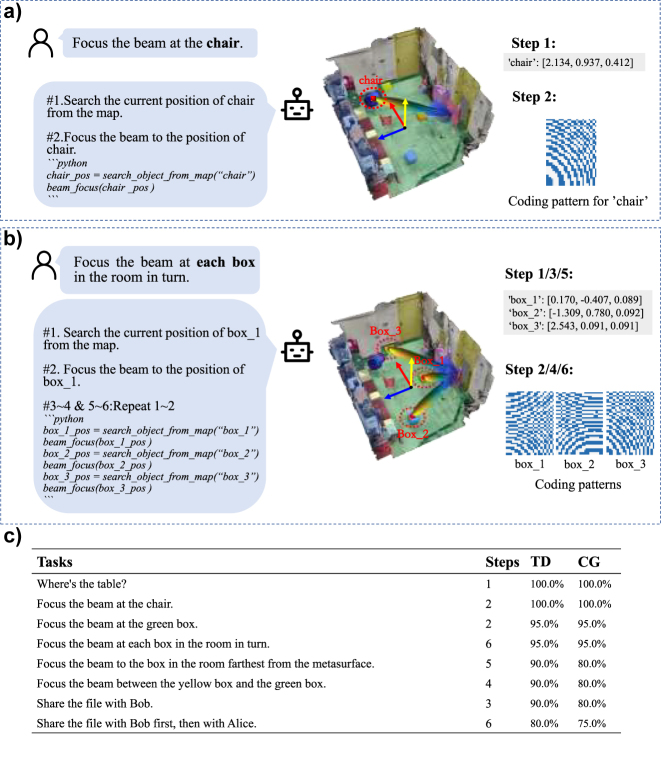
Experimental results of autonomous task decomposition. (a) When a user sends a request in a linguistic way, the agent can decompose the task based on LLM and then generate the execution code corresponding to each step, which is executed one by one. For instance, when user command is “focus the beam on the chair”, and the system decomposes the task into two steps: the first step is to search for the location of the “chair” from the semantic map, and the second step is to focus the beam on that location. (b) Another more complex task with the same steps as (a). (c) The success rates for 8 different EM manipulations tasks with different complexities in performing the task decomposition (TD) and code generation (CG) over 100 repetitions for each task.

In this section, we would like to examine the self-planning capability of programmable metasurface agent for relatively complex EM manipulation tasks. To this end, we have explored so-called zero-sample learning strategy of LLMs, conducted a set of tests with the GPT-3.5-turbo LLM model released by OpenAI, and loaded the pre-established 3D visual-semantic map of environment into LLM in form of prompt to enable LLM to get acquittance with the surrounding environment. See examples about the prompt project in Supplementary Note 2. Figure 3a reports a relatively simple case where a user asks the programmable metasurface agent to focus radiation beam towards a chair through the text input, and the agent will generate the corresponding Python codes and execute them step by step. In particular, the agent will decompose the original ambiguous request into two implementable subtasks: (i) searching for the location of the chair in the visual semantic map, (ii) focusing the radiation beam towards the intended chair. When the host computer completes the code generation, it will generate the control coding sequences of programmable metasurface for the intended beam manipulation, and the MCU will control the programmable metasurface to manipulate the radiation beam. We have conducted another set of more complex EM manipulation task, and reported the results in Figure 3b, where our major concern is to examine the generalizability of comprehension and ambiguous language instructions. To this end, the user does not explicitly specify the object on which the beam needs to be directed, but rather requests that it be focused towards each box in turn. We have selected 8 sets of user command tasks to test the developed programmable metasurface agent, and repeated each set of tasks 100 times and recorded the success rate of task decomposition and code generation in Figure 3c. It can be observed that the programmable metasurface agent is capable of understanding the user’s request and completing the task successfully. Note that the success rate of task completion decreases gradually, but it still can meet the demand, as the complexity of the task increases. We can see from above results that the programmable metasurface agent has the self-planning capability in the sense of decomposing a relatively ambiguous EM manipulation task into a sequence of executable tasks.

### 3.3 Results of exploring the unknown environment

**Figure 4:**
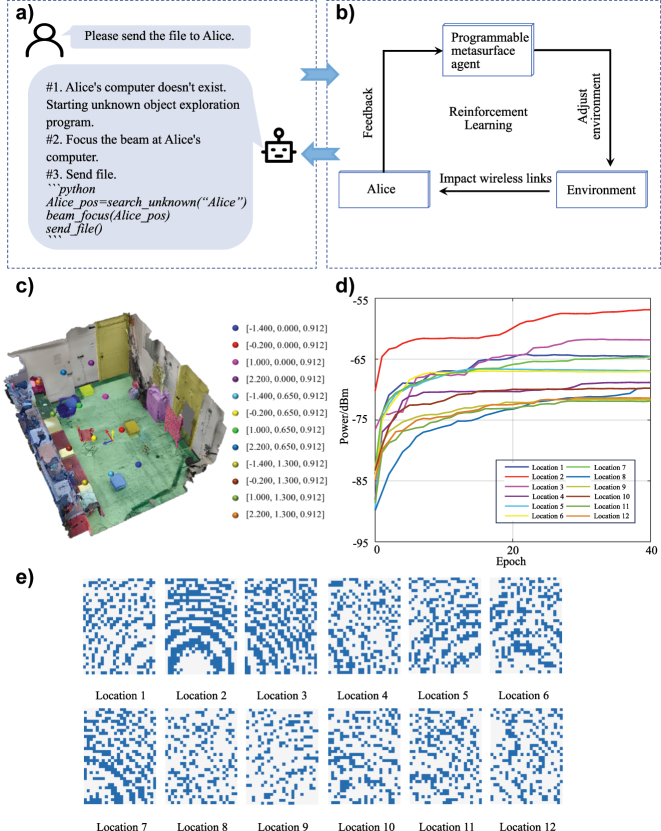
Experimental results of programmable metasurface agent in exploring unknown environments. (a) Task decomposition, (b) system diagram, (c) the location of the receiver in our experiment, (d) the power of Bob’s received signal as a function of learning epochs, (e) the optimized control coding patterns of programmable metasurface.

Note that we have implicitly assumed that the surrounding physical environment, i.e., wireless channel, has been known for the programmable metasurface agent above, and the control coding pattern of progammable metasurface can be explicitly obtained in advance once the intended user is localized. However, in most of practical applications, the surrounding environment is usually hard to be estimated in advance for progammable metasurface agent, especially when the location of the target user is inaccessible, for example, if there are obstacles between the target and metasurface. To resolve this issue, we here would like to utilize the reinforcement learning-based online search algorithm in Ref. [33] to automatically adjust the control coding pattern through the active interaction with the unknown environment. As we show in Figure 4a and b, the programmable metasurface agent takes a so-called trial-and-error approach to adjust its coding pattern according to the feedback from the surrounding environment. In particular, the programmable metasurface agent runs a three-layer artificial neural network to make decision to adjust its control coding pattern, and correspondingly changes the EM field distribution of the unknown environment and the signal intensity received by the user. After that, the user will measure its received signal strength, and feedback the measurement result to the agent to generate reward function for parament updating of the artificial neural network. The detailed learning process could be summarized as follows: an epoch consists of 384 steps, in every step the neural network outputs the probability distribution of state reversal for each unit according to the current coding state to guide the programmable metasurface to switch its coding pattern, then records the decision trace and feedbacked rewards for policy updates. At the end of each epoch, the agent will update its policy *Θ* with policy gradient method as we show in Eq. (1). Here, we use *a* and *s* to denote actions and states. The learning rate *α* is set to 0.001, the discounted factor *γ* is set to 0.99, and the single-step reward function is generated from the received signal power of the user according to Eq. (2).

(1) $$\Theta^{*}\leftarrow \Theta +\alpha \sum_{l=1}^{384}\sum_{i=l}^{384}\gamma^{i-l}\text{rewar}d_{i}\nabla \text{log}p_{\Theta }\left(a_{l}|s_{l}\right)$$

(2) $$\begin{array}{ll} \text{rewar}d_{i} & =\left(\text{powe}r_{i}-\text{powe}r_{i-1}\right)\\ & \;\times abs\left(\text{powe}r_{i}-\text{powe}r_{i-1}\right) \end{array}$$

We have conducted a set of experiments to verify our system’s capacity of exploring the unknown environment. The signal intensity as a function of learning epochs has been plotted in Figure 4d, and corresponding optimized coding patterns have been shown in Figure 4e. In our experiments, we respectively place the user in different unknown locations to verify the effectiveness and stability of our reinforcement learning-based online search algorithm, and successfully improve the power gain in a factor of above 10 dB. From this set of experimental results, we can demonstrate that the developed programmable metasurface agent is capable of autonomously exploring the unknown environment and online optimizing its control coding pattern to improve the communication’s quality.

### 3.4 Results of wireless information sharing

**Figure 5:**
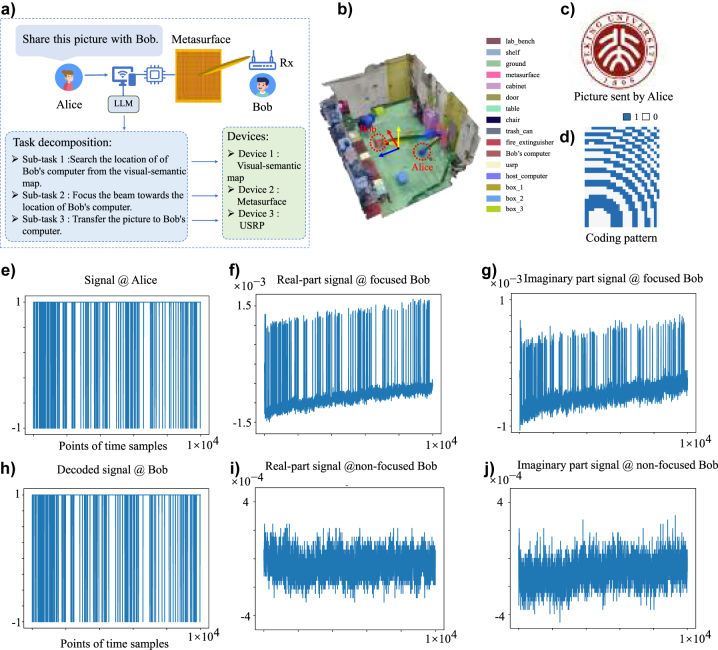
Experimental results of wireless information sharing with the programmable metasurface agent. (a) Operation procedure and task decomposition, (b) 3D visual-semantic map, (c) the RGB picture transmitted from Alice to Bob, (d) the control coding pattern of programmable metasurface, (e) A 10^4^-length signal sent from Alice, (f–g) the real and imaginary parts of the signal received by Bob when the beam is focused towards Bob using programmable metasurface. (h) The decoded signal at Bob corresponding to that of (e), (i and j) the real and imaginary parts of the signal received by Bob when the focusing beam is not available.

Here, we will consider a more realistic application using the programmable metasurface agent, i.e., wireless information sharing, as shown in Figure 5a and b, where Alice wants to share an RBG picture of logo of Peking university (see Figure 5c) to Bob in our lab environment. To that end, Alice uploads the picture to the host computer along with a text request “Please share this picture with Bob”. When receiving this request, the programmable metasurface agent will decompose the ambiguous request into three executable subtasks, and sequentially dispatches these subtasks to the 3D visual-semantic map, metasurface and USRP, as detailed in Figure 5a. After receiving the subtask from the LLM, the agent will recognize and localize Bob and exports the Bob’s location to the programmable metasurface through the 3D visual-semantic map. Then, the agent adapts the control coding pattern (see Figure 5d) in terms of Bob’s location such that its radiation beam can be well focused toward Bob in a remarkably enhanced signal’s intensity. Thereby, the picture to be shared can be transmitted from Alice to Bob by the USRP with the wireless communication link enhanced by the programmable metasurface. The first 10,000 sampling points of raw signal received by Bob has been shown in Figure 5f and g. After some straightforward signal process, the decoded signal at Bob is able to be successfully recovered with a low bit error rate of 0.179 %, as shown in Figure 5h, implying that the programmable metasurface agent can successfully accomplish the information sharing task. Moreover, in order to highlight the importance of programmable metasurface in the wireless information sharing task, we have reported the corresponding first 10,000 sampling points of Bob’s received signal when the programmable metasurface is not available for the beam focusing in Figure 5i and j. We can see that the SNR (signal-to-noise) of Bob’s received signal with the programmable metasurface is significantly higher than that without the programmable metaurface, which means the programmable metasurface can significantly improve the wireless link with the target user and thus plays an important role in the wireless information sharing. Now, we can conclude from above primary experimental results that the developed programmable metasurface agent, with the reasoning and understanding ability of LLM and the beam manipulation capacity of programmable metasurface, is able to intelligently understand and decompose information sharing tasks given in natural language forms and automatically schedule its sub-devices to execute corresponding subtasks.

## 4 Conclusions

We have presented the concept of language-controllable programmable metasurface, i.e., programmable metasurface agent, by combining the power of LLMs in attainting human-level intelligence with the flexible EM manipulation capability of programmable metasufaces. We have established a proof-of-principle programmable metasurface agent, and demonstrated experimentally the strength of programmable metasurface agent in three aspects: (1) the self-planning capability in performing complex EM manipulation tasks; (2) exploring unknown environments and continuously improving the EM manipulation through trial and error according to the feedback from surrounding environments; (3) sharing information and collaborating with other agents or real people. We expect that the proposed strategy could hold promising potential in advancing programmable metasurfaces towards human-level autonomous agents.

## Supplementary Material

Supplementary Material Details
