# Supplementary material for: Language-controllable programmable metasurface empowered by large language models
Source: Nanophotonics. 2024 Jan 4;13(12):2213–22. doi: 10.1515/nanoph-2023-0646 (PMC11501434; doi:10.1515/nanoph-2023-0646)
Supplement: Supplementary file 1 — Supplementary Material Details [file j_nanoph-2023-0646_suppl_001.pdf]

Language-controllable programmable metasurface empowered by large language models

Shengguo Hu[1+], Jiawen Xu[1+], Mingyi Li[1], Tie Jun Cui[2,3] and Lianlin Li[1,3]

[1] State Key Laboratory of Advanced Optical Communication Systems and Networks, School of Electronics, Peking University, Beijing 100871, China

[2] State Key Laboratory of Millimeter Waves, Southeast University, Nanjing 210096, China

[3] Pazhou Laboratory (Huangpu), Guangzhou, Guangdong 510555, China

Supplementary Note 1. Design of Programmable Metasurface

The designed programmable metasurface works around 5.5 GHz. The programmable metasurface is composed of independently-controllable 32×24 meta-atoms. Since each meta-atom has a size of $24.4\times24.4mm^2$, the whole metasurface has size of $0.780\times0.585m^2$ in total. We remark that the whole metasurface is composed of 3×4 identical panels due to the restriction of fabrication, and each panel has 8 x 8 meta-atoms.

The meta-atom is composed of two substrate layers: the top substrate is F4B with the relative permittivity of 2.44 and loss tangent of 0.0019, and the bottom substrate is FR4. The top square patch, which is responsible for reflecting incoming electromagnetic (EM) waves, is integrated with a SMP1345-079LF PIN diode connected to the ground plane via a hole. We choose the SMP1345-079LF diode because it has relatively low insertion loss (< 0.2dB) and high isolation>13dB) in the desired frequency band. A TDK chip inductor with inductance L = 33nH (MLK1005S33NJT000) is used to suppress the AC coupling to ground. We examine the EM performance of the electronically-controllable meta-atom numerically and experimentally. In numerical simulations, we use a commercial full-wave EM simulator, CST Microwave Transient Simulation Package 2018. Additionally, a series lumped-parameter circuit is deliberately chosen to model the PIN diode. When the diode is switched ON, it is represented by a 0.7nH inductor in series with a 2Ω resistor. By contrast, when the diode is switched OFF, it is modeled by a 1.8pF capacitor in series with a 0.7nH inductor. The meta-atom has been designed, fabricated and tested. The simulation and experiment results are compared in **Figure 1(e)**. We observe that the reflection phase of the meta-atom experiences 180° phase difference when the PIN diode is switched from ON (OFF) to OFF (ON) in the frequency range 5.48-5.66 GHz. The phase change can be accomplished by switching the external DC voltage applied to the PIN diode from 12V to 0V.

The whole programmable metasurface is electronically controlled with a FPGA-based Micro-Control-Unit (MCU), as shown in the insert of **Supplementary Figure 1**. A FPGA chip is used to distribute all commands to 768 PIN diodes. To achieve the real-time and flexible controls of 768 PIN diodes soldered in the programmable metasurface, a MCU with size of $90\times90mm^2$ is designed and assembled on the upper rear of the metasurface. This MCU is connected with three metasuface panels through three 0.3m-long winding wires in parallel, each of which is connected with another two metasurface panels in series, as illustrated in **Supplementary Figure 1**. The MCU is

responsible for dispatching all commands sent from a master computer subject to one common clock (CLK) signal. In our work, the adopted CLK is 50MHz, and the switching time of PIN diode is about 2.5us each cycle. Each metasurface panel is equipped with eight 8-bit shift registers (SN74LV595APW), and every 8 PIN diodes share the same shift register. With the use of shift registers, 8 PIN diodes are sequentially controlled. Then MCU will send the commands over 24 independent branch channels, leading to almost real-time manipulations of all PIN diodes. In addition, 768 green-color LEDs are soldiered to indicate the status of the associated PIN diodes, in particular, to indicate clearly whether the PIN diode works well or not. We remark that the proposed control strategy can be readily extended for more PIN diodes by concatenating more metasurface panels, allowing adjustable rearrangement of metasurface panels to meet various needs.

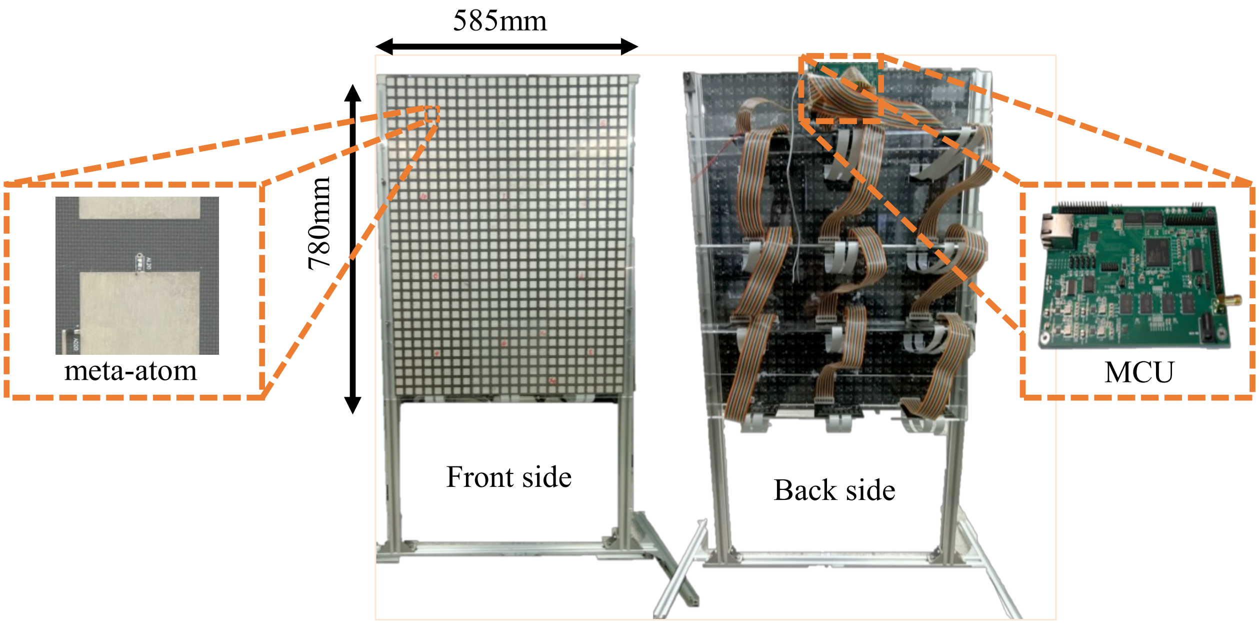

Supplementary Figure 1 | The pictures of the designed programmable metasurface works around 5.5 GHz, where the front- and back-view pictures are provided. In this set of figures, the designed meta-atom and FPGA-based micro control unit (MCU) are inserted in figure, respectively.

Supplementary Note 2. LLM's prompt

Our programmable metasurface agent system uses the following prompt project content:

Prompt:

#01 You are an AI agent based on the programmable metasurface that can perform task inference and task decomposition based on user inputs based on the semantic map information and basic

function information that I have provided you with, and write python code to control the programmable hypersurface to accomplish the corresponding tasks.
#02 You are not to use any other hypothetical functions that you think might exist.
#03 You can use simple Python functions from libraries such as math and numpy.
#04 Do not import python libraries or defining functions
#05 Never make assumptions.
The following objects are in the semantic map, and you are to refer to them using these exact names:
"""
    << *SemanticMap* >>
"""
Here are the basic functions that you can use to command the programmable metasurface:
"""
    << *BasicFunction* >>
"""

For different environments, you can output the corresponding semantic map according to the following format, and then input the names of all the objects contained in the semantic map at << *SemanticMap* >>:

"""
'chair_1','room', 'corridor', 'lab_bench', 'table', 'shelf', 'chair', 'yellow_box', 'green_box', 'blue_box', 'cabinet_1', 'cabinet_2','metasurface_2.4G', 'metasurface_5.5G', 'metasurface_9.7G', 'meter_box', 'door_1', 'door_2', 'box_1', 'box_2', 'box_3', 'trash_can', 'oscilloscope', 'usrp', 'host_computer_1', 'host_computer_2','student_power_1', 'student_power_2', 'student_power_3', 'Alice_computer', 'Bob_computer', 'fire_extinguisher', 'big_vehicle', 'Wooden_block'
"""

The << *BasicFunctions* >> used in our system are as follows:

"""
search_object_from_map (object_name): Takes a string as input indicating the name of an object to be searched for, and returns a list of 3 floats indicating its X,Y,Z coordinates.
beam_focus (position) - Focus the beam at the position specified as a list of three arguments corresponding to X, Y, Z coordinates.
search_unknown (object_name) - Takes a string as input indicating the name of an object to search in an unknown environment.
send_file() - Send the specified file through the programmable metasurface.
"""
A few useful things:
Instead of *FocusBeamToPositionAsync()* or *FocusBeamToZAsync()*, you should use the

function *beam_focus()* that I have defined for you.
